# Supplementary material for: Heat flows solubilize apatite to boost phosphate availability for prebiotic chemistry
Source: Nat Commun. 2025 Feb 20;16:1809. doi: 10.1038/s41467-025-57110-3 (PMC11842809; doi:10.1038/s41467-025-57110-3)
Supplement: Supplementary file 1 — Supplementary Information [file 41467_2025_57110_MOESM1_ESM.pdf]

Supplementary information for

Heat flows solubilize apatite to boost phosphate availability for prebiotic chemistry

Thomas Matreux[1,7,+], Almuth Schmid[1,+], Mechthild Rappold[1],
Daniel Weller[2], Ayşe Zeynep Çalışkanoğlu[2], Kelsey R. Moore[3,4],
Tanja Bosak[4], Donald B. Dingwell[2], Konstantin Karaghiosoff[5],
François Guyot[6], Bettina Scheu[2], Dieter Braun[1], Christof B. Mast[1,#]

[1]Systems Biophysics, Ludwig Maximilians University, Munich, Germany
[2]Earth and Environmental Sciences, Ludwig Maximilians University, Munich, Germany
[3]Department of Earth and Planetary Sciences, Johns Hopkins University, Baltimore, MD, USA
[4]Department of Earth, Atmospheric and Planetary Sciences, Massachusetts Institute of Technology, Cambridge, MA, USA
[5]Department of Chemistry, Ludwig Maximilians University, Munich, Germany
[6]Institut de Minéralogie, de Physique des Matériaux et de Cosmochimie (IMPMC), MNHN, CNRS, IRD, Sorbonne Université, Paris, France
[7]Present address: Laboratoire de Biophysique et Evolution, UMR CNRS-ESPCI 8231 Chimie Biologie Innovation, PSL University, Paris, France

[+] These authors contributed equally to this work.
# Corresponding author: christof.mast@physik.uni-muenchen.de

Supplementary Figures 3
Supplementary Figure 1. Comparison of the apatite samples in this study. 3
Supplementary Figure 2. Characterization of leaching of natural apatites. 4
Supplementary Figure 3. Differential accumulation of calcium and phosphate from acidic-dissolved apatite. 5
Supplementary Figure 4. Precipitation under different temperatures and pH. 6
Supplementary Figure 5. Precipitates after re-neutralization. 7
Supplementary Figure 6. Experimental setup and partitioning of the heat-flow microfluidics...... 8
Supplementary Figure 7. SEM images of geomaterials tested for phosphate leaching and phosphate polymerization Part 1 9
Supplementary Figure 8. SEM images of geomaterials tested for phosphate leaching and phosphate polymerization Part 2 10
Supplementary Figure 9. Numerical simulation of long-time heat-flow-driven phosphate accumulation using Comsol. 11
Supplementary Figure 10. Polymerization of phosphate on geomaterial. 12
Supplementary Figure 11. Detection of polyphosphates 13
Supplementary Tables 14
Supplementary Table 1. CaO/P_2O_5 ratios of precipitates in re-neutralized samples. 14
Supplementary Table 2. Composition of geomaterials tested for phosphate leaching and phosphate polymerization. 15
Supplementary references 16

Supplementary Figures

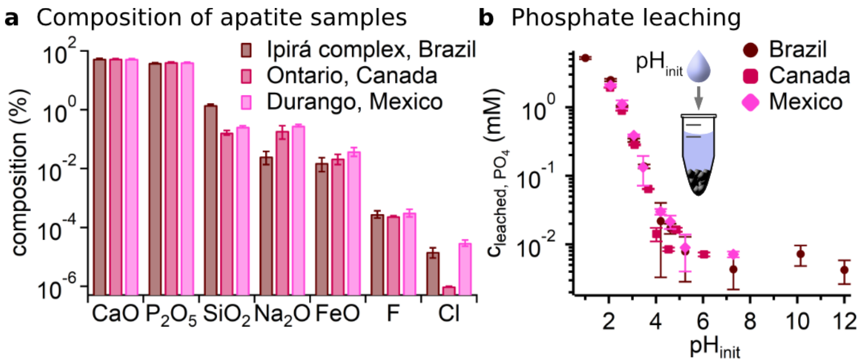

Supplementary Figure 1. Comparison of the apatite samples in this study.

(a) Oxide composition obtained by XRF analysis, see also main text Table 1. **(b)** Leached phosphate concentration over a pH range from 1 to 12, for full compositions of leachates see Supplementary Fig. 2a-c. Despite the different origins and compositions of the apatites used, concentrations profiles are similar. All error bars indicate the SD.

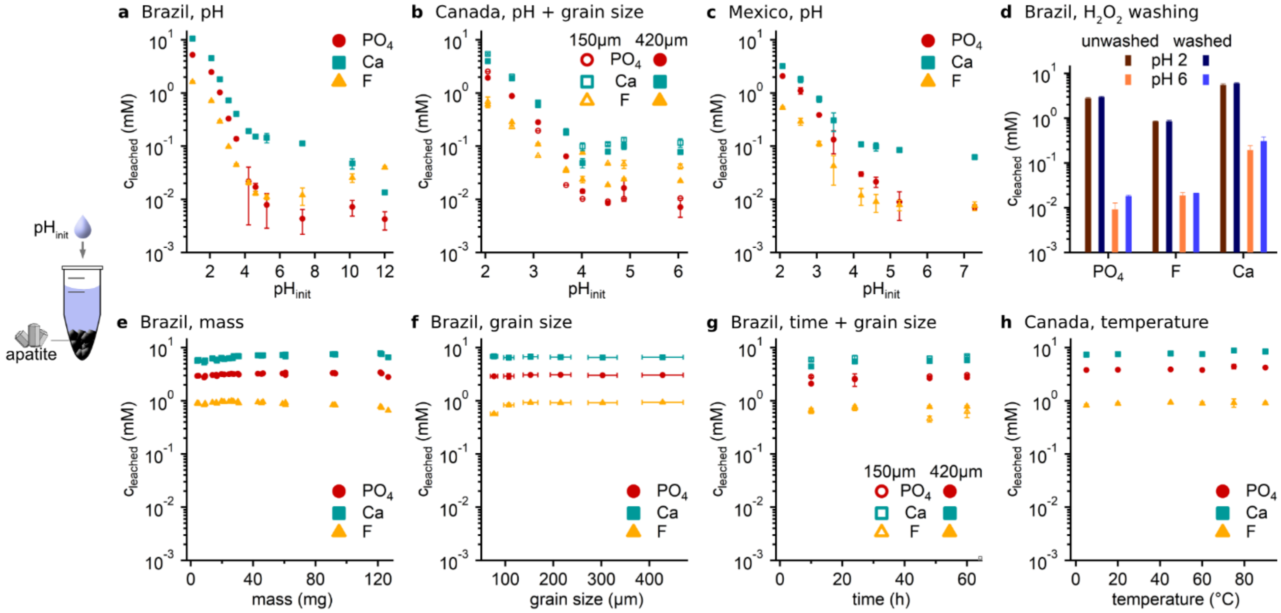

Supplementary Figure 2. Characterization of leaching of natural apatites.

Leaching was done for 60 h (if not stated otherwise) at 60 °C (if not stated otherwise) and for 30 mg of grains of diameter 355-500 µm in 150 µl of ion chromatography water. After the experimental time, ion concentrations were analyzed using ion chromatography (see Methods). All experiments were done in triplicate with error bars indicating the standard deviation. **(a-c)** Different natural apatites (for compositions, see main text Table 1) were leached at various initial pH values. Concentrations and their ratios show variation over pH but remain similar for the three different apatites. **(d)** Effect of H_2O_2-washing (10% vol.) on leached concentrations compared to unwashed grains yields comparable concentrations of the fluorapatite-forming ions fluorine, calcium and phosphate. **(e-h)** Neither mass-to-volume ratio nor temperature, grain size, or time changes leached concentrations and ratios between species. All error bars indicate the SD.

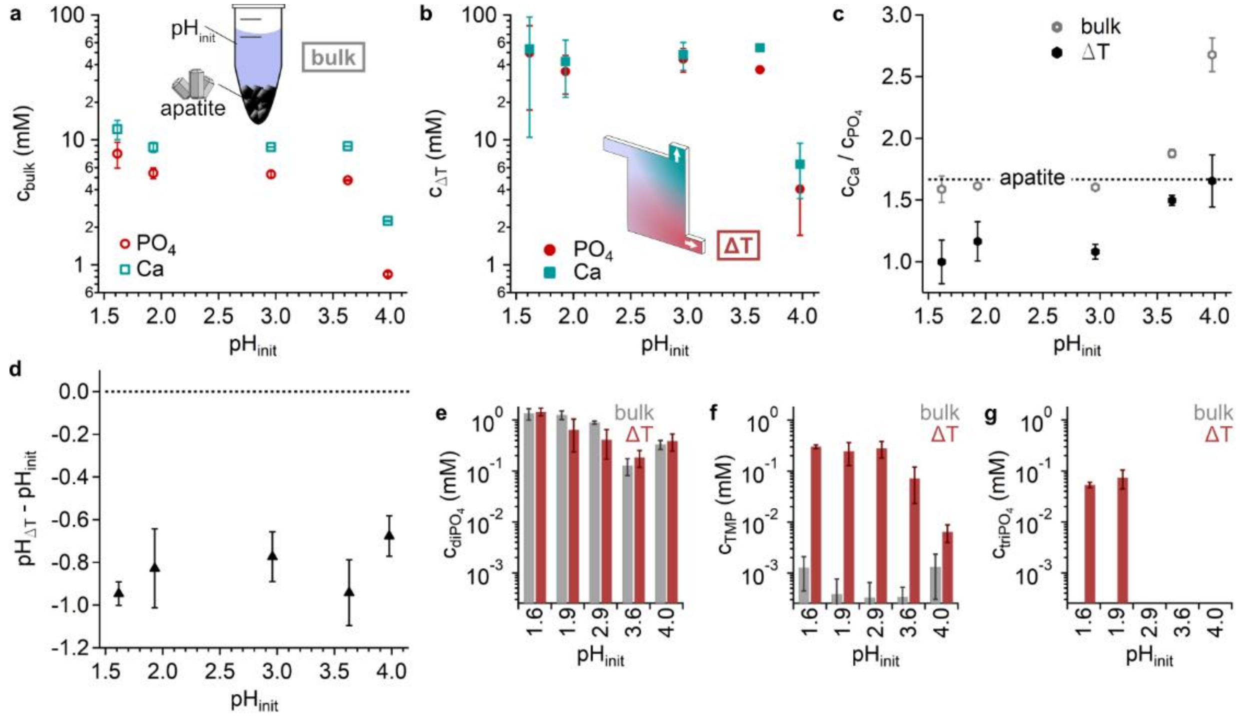

Supplementary Figure 3. Differential accumulation of calcium and phosphate from acidic-dissolved apatite.

(a) Apatite samples (Brazil, see main text Table 1) were acidic-dissolved by repeated addition of HCl (see Methods). When equilibrium was reached, and the pH stayed constant, samples were diluted with three fractions of water, previously adjusted to the same pH. Then, ion composition was measured using IC, showing the pH-dependent dissolution profile. **(b)** Flow through a microfluidic chamber that was exposed to a temperature gradient accumulates both phosphate and calcium but with different strengths. **(c)** Thereby, the initial 5:3 Ca:PO_4 ratio is altered to 1:1. **(d)** This ionic change is balanced by a shift in pH, thus keeping local charge neutrality. **(e-g)** Moderate heating to 180 °C triggered the formation of condensed phosphates such as trimetaphosphate (TMP, f), shown for pH neutralized bulk (grey) and heat-flow-altered solutions (ΔT, red). All error bars indicate the SD.

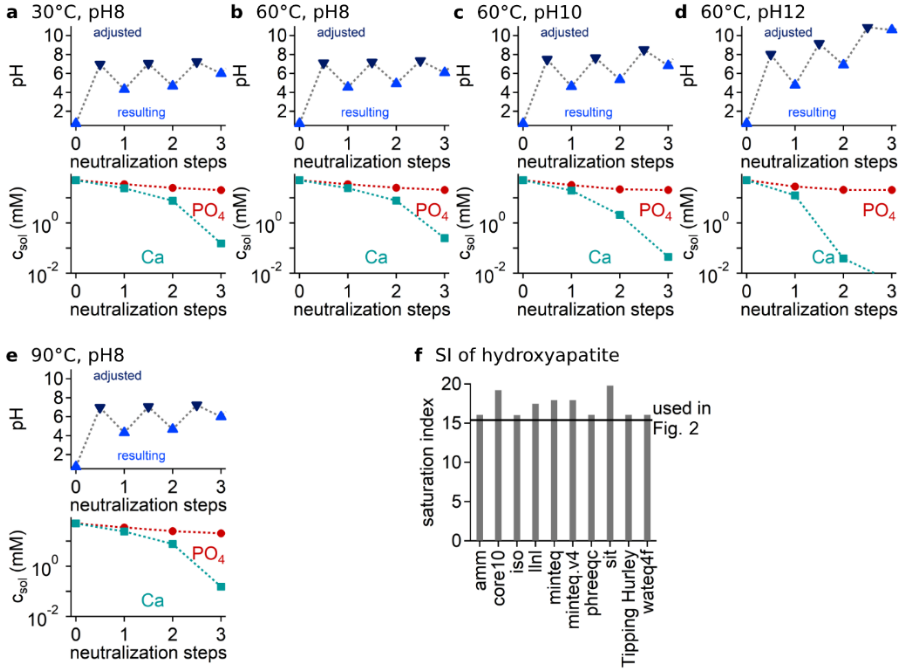

Supplementary Figure 4. Precipitation under different temperatures and pH.

(a-e) In the experiments, we incubated samples for precipitation at 60 °C and set pH 8 in each step. Different temperatures and pH values did not change the dynamics; only the final pH values were modified. **(f)** Starting from a composition (50 mM P as PO_4, 50 mM Ca, 20 mM Cl (measured via IC, from acidic dissolution) and addition of 300 mM NaOH, see Methods) as in Fig. 1, we compared different databases for the saturation index (SI = log(IAP/K_{sp})) with IAP the ion activity product and K_{sp} the solubility product) of hydroxyapatite. Results were very similar and validated our method, which shows slightly inferior saturation indices.

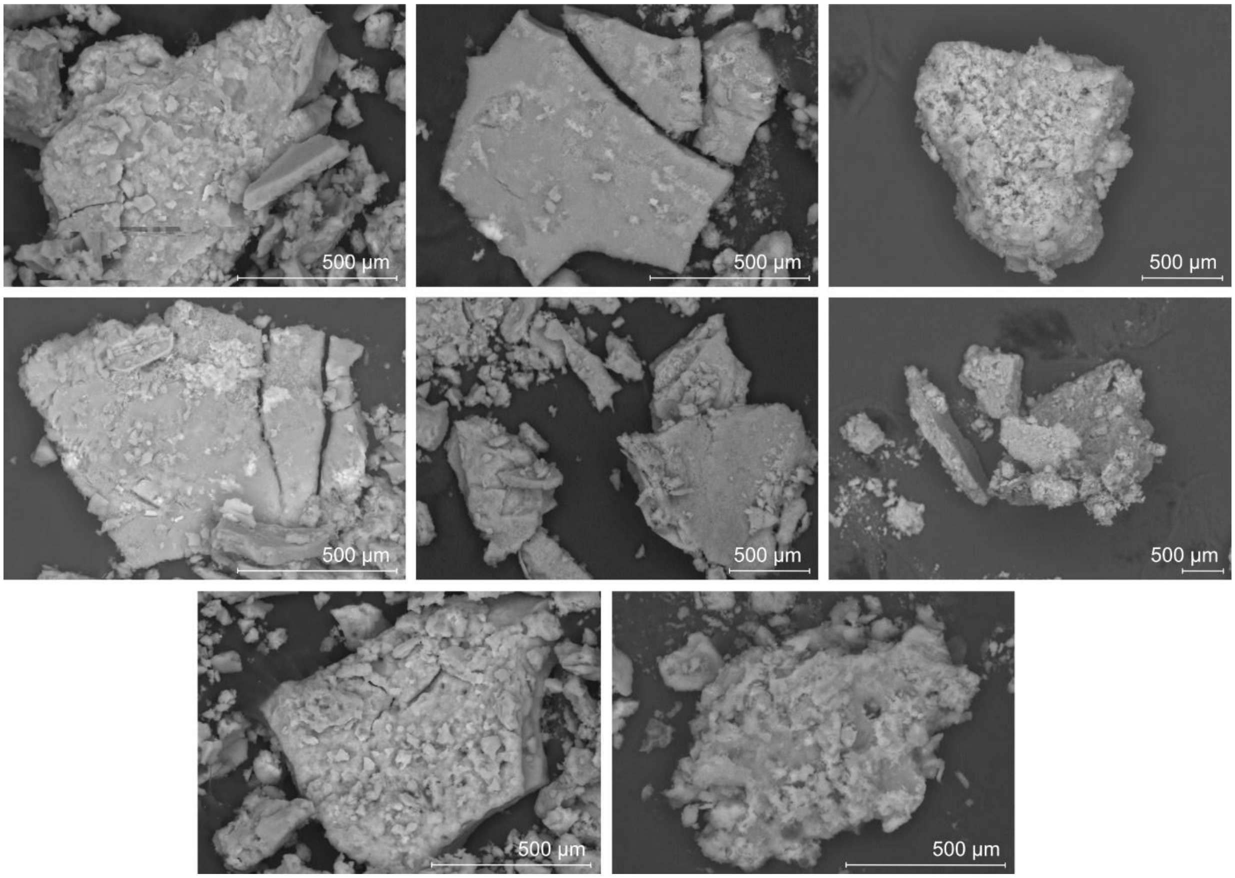

Supplementary Figure 5. Precipitates after re-neutralization.

Precipitation of solutions upon pH neutralization under SEM (see Methods) , $CaO:P_2O_5$ ratios are given in Supplementary Table 1.

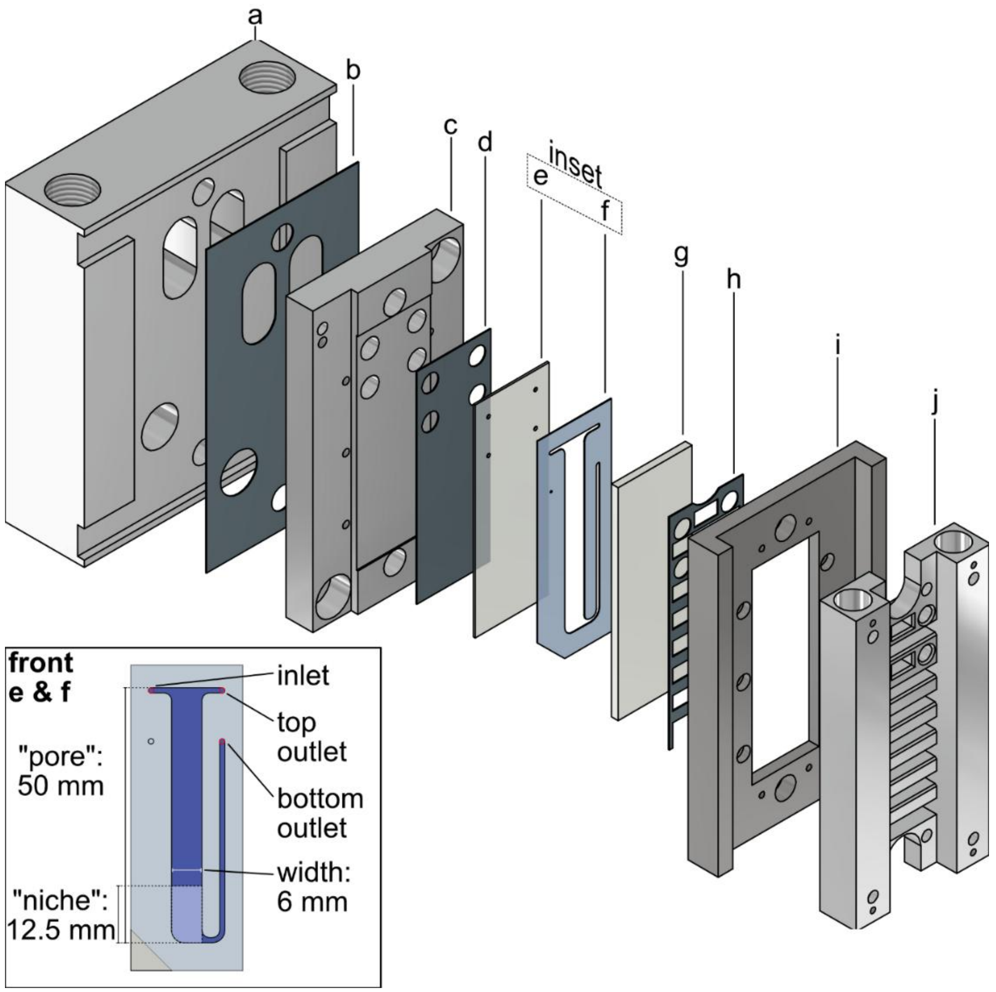

Supplementary Figure 6. Experimental setup and partitioning of the heat-flow microfluidics. The microfluidic structure (f) of the heat flow chambers is cut from 0.2 mm thick FEP film using an industrial plotter and sandwiched between a 0.5 mm (e) and a 2 mm (g) thick sapphire. The backside sapphire (e) is in contact with an aluminum element (c) which, like the sapphire, has four access holes with a diameter of 1 mm each through which the heat flow chamber can be filled and at which the continuous flow, as used for the experiments in main text Fig. 2 and 3, is applied. The aluminum element (c) in turn is placed on a heat exchanger made of aluminum (a), to which a temperature cryostat is connected for cooling. On the other side, the 2mm sapphire (g) is held by a steel frame (i), which is connected to (c) by screws with a defined torque, thus sealing the microfluidics (e-g). An aluminum frame (j) is mounted on the steel frame, which contains two electric heating cartridges, each with a heating capacity of 160 W. The aluminum frame is in contact with the sapphire (g), thereby heating it. The heat transfer is optimized by graphite foils (b, d, h). Inset: The microfluidic structure (f) consists of a 50 mm high, 6 mm wide and 0.2 mm thick chamber (“pore”), which is connected to the access holes via channels (e.g., the inlet channel is connected to the solution named “bulk” in main text Fig. 2-3). The “niche” defined in main text Fig. 3 corresponds to the lowest quarter of the chamber, i.e. 12.5 mm high.

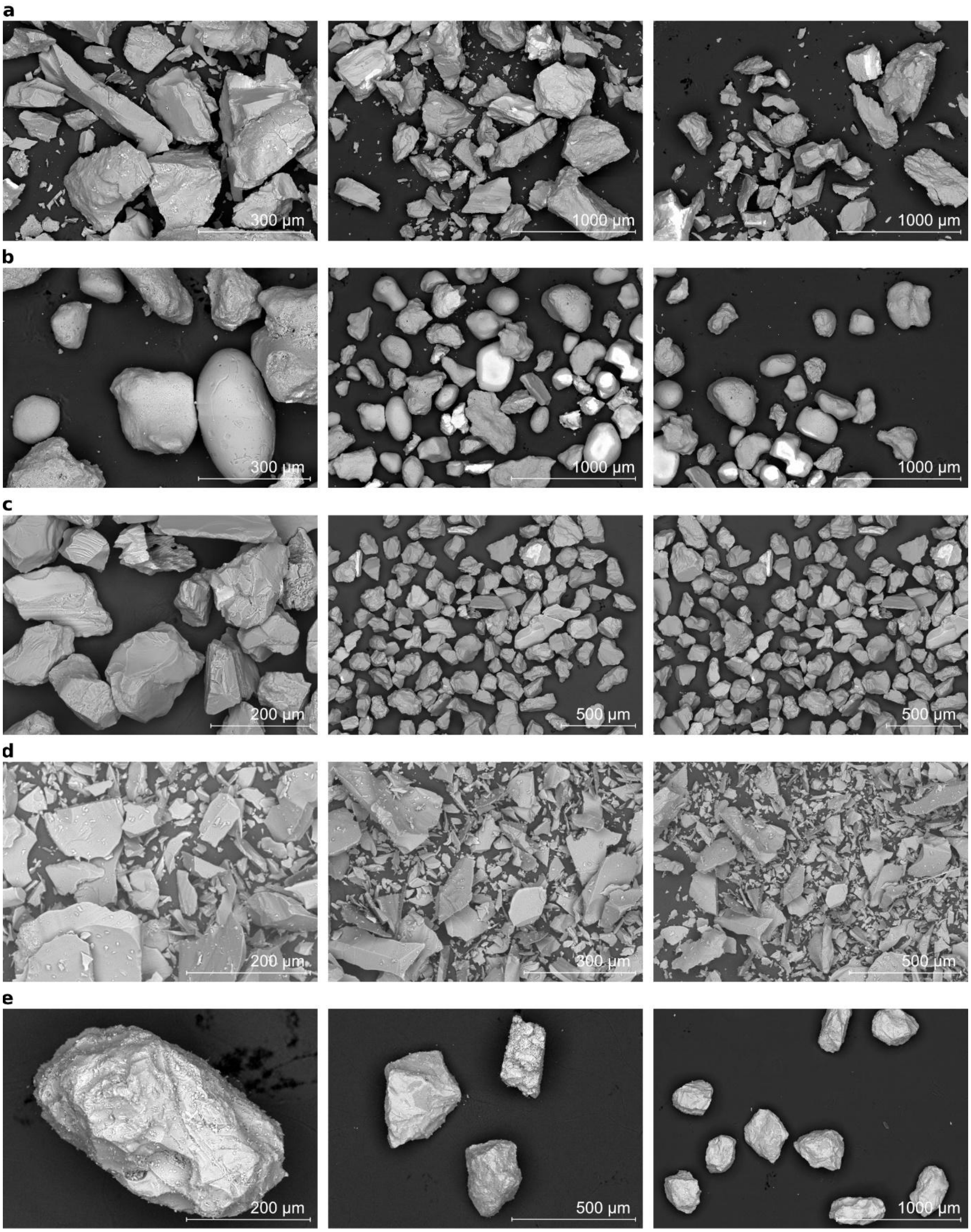

Supplementary Figure 7. SEM images of geomaterials tested for phosphate leaching and phosphate polymerization Part 1.

Compositions are given in Supplementary Table 2. **(a)** BSS: Basalt sand, **(b)** CAS: Carbonate sand, **(c)** SCS: Siliciclastic sand, **(d)** VCG: Volcanic glass, **(e)** BF2: Basalt F2

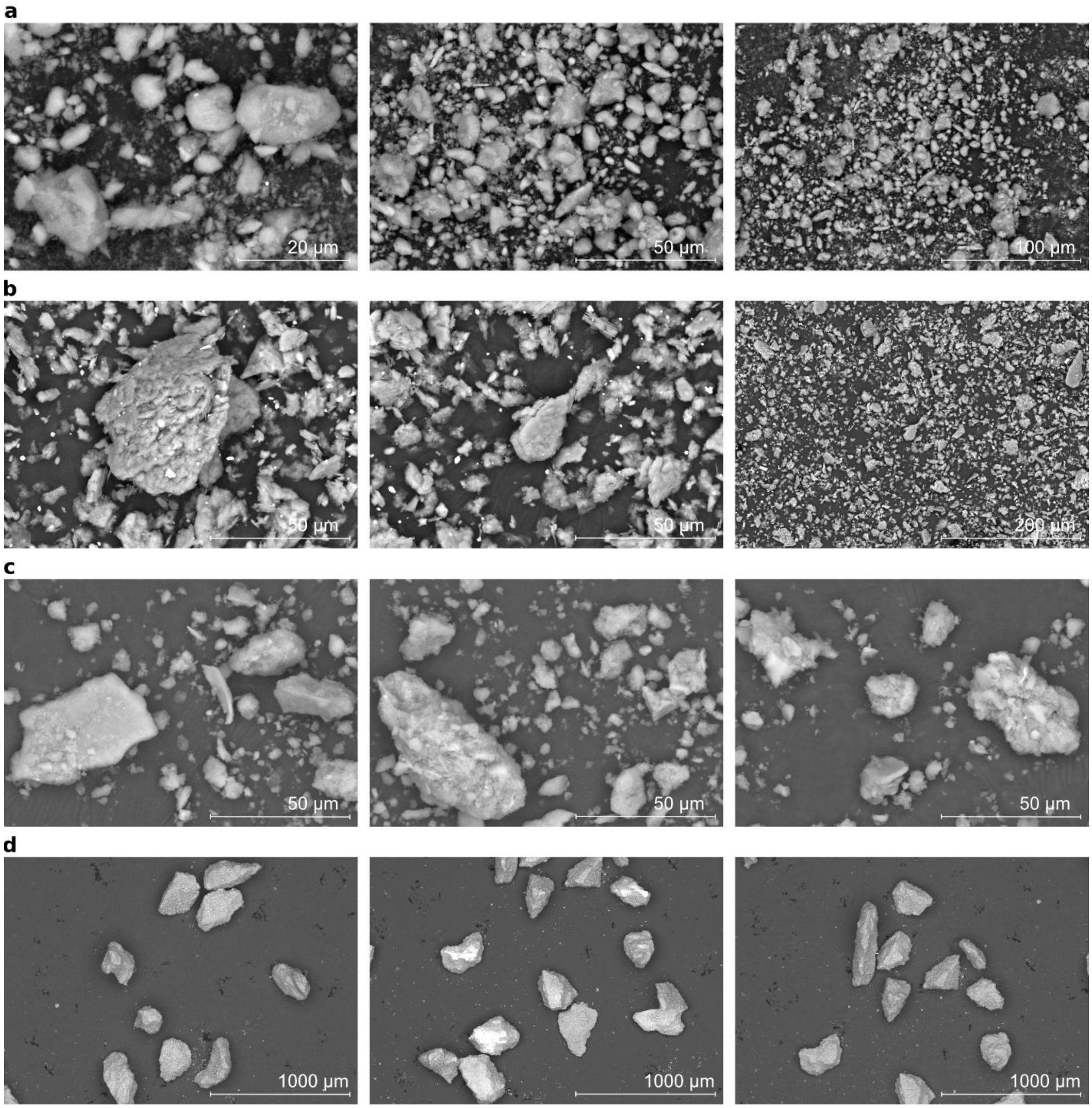

Supplementary Figure 8. SEM images of geomaterials tested for phosphate leaching and phosphate polymerization Part 2.

Compositions are given in Supplementary Table 2. **(a)** ILL: Illite, **(b)** KAO: Kaolinite, **(c)** MON: Montmorillonite, **(d)** ZEO: Zeolite

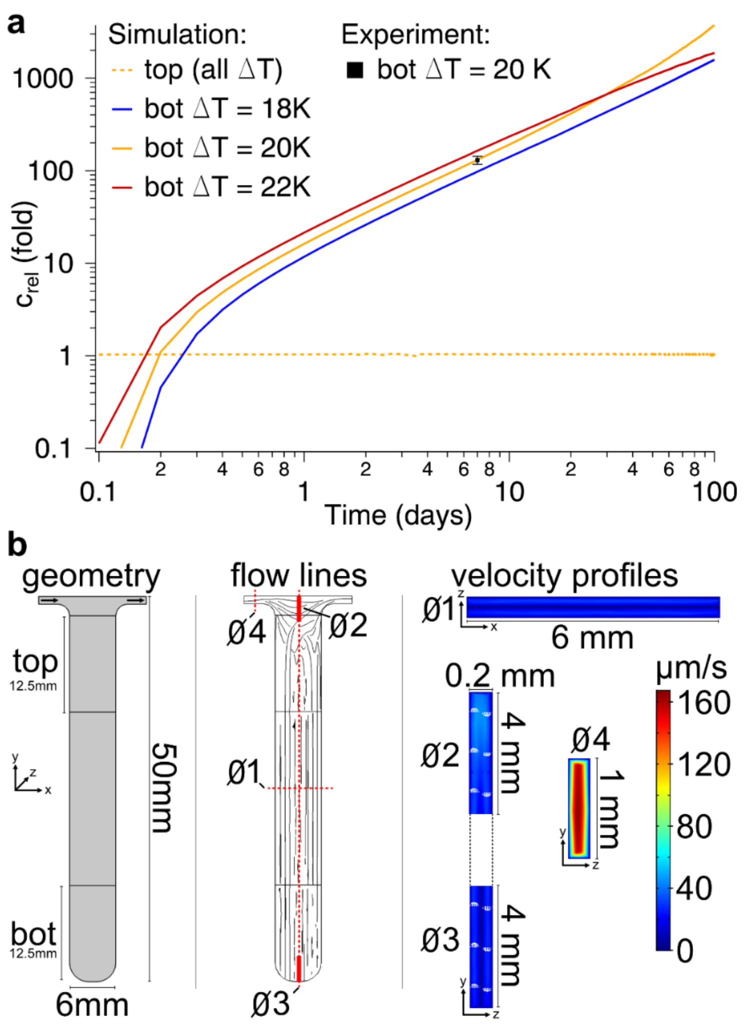

Supplementary Figure 9. Numerical simulation of long-time heat-flow-driven phosphate accumulation using Comsol.

(a) The geometry of the simulation corresponds to the setup in Supplementary Fig. 6. The flow rate through the upper left inlet and upper right outlet was set to 30 nl s^{-1}, and the temperature difference across the 0.2 mm thick chamber was varied between 18 K, 20 K and 22 K, showing an exponential dependency, as expected[1]. For the phosphate ions, a diffusion coefficient of D = 824 µm^2 s^{-1} and a Soret coefficient S_T = 0.027 K^{-1} were used[2]. A 1000-fold phosphate concentration is reached after about 100 days. The positions "top" and "bot" (bottom) and flow direction are defined in b) on the left. **(b)** The center shows the flow lines of the solvent, which are dominated by the external flow in the uppermost part of the chamber and by thermal convection in the rest of the chamber. On the right side, the velocity profiles at cross-sections 1 (convection, maximum velocity approx. 28 µm s^{-1}), 2 (upper chamber part, y-z axis, 4mm long, maximum velocity approx. 40 µm s^{-1}), 3 (lower chamber part, y-z axis, 4 mm long, maximum speed approx. 28 µm s^{-1}) and 4 (at the inlet, in which the flow rate of 30 nl s^{-1} generates a maximum speed of approx. 160 µm s^{-1}) are shown. The simulation setup was analogous to previous studies[3].

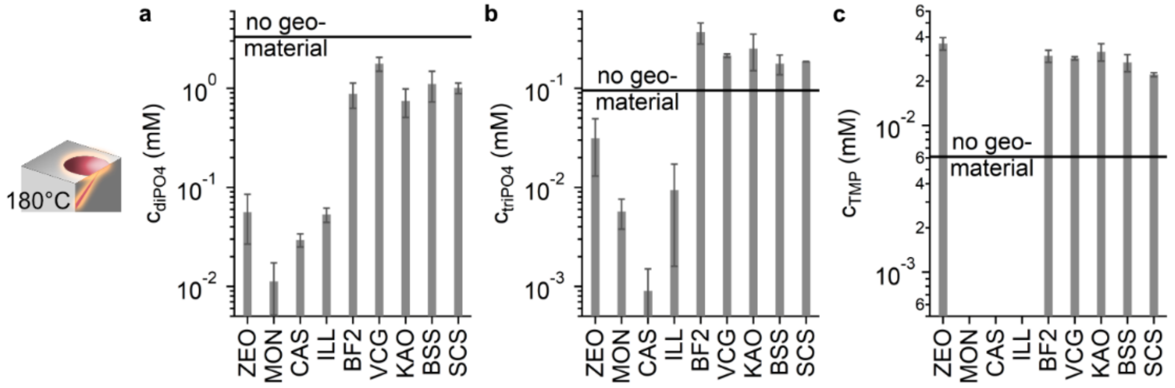

Supplementary Figure 10. Polymerization of phosphate on geomaterial.

10 µl of 10 mM NaH_2PO_4 adjusted to pH 7 were added to 30 mg of geomaterial in glass vials and heated at 180 °C for three days. Analysis of redissolved phosphate species **(a-c)** shows clear positive or negative influence of geomaterial surface, which proved to be especially beneficial for the formation of trimetaphosphate (TMP, **c**). ZEO: Zeolite, MON: Montmorillonite, CAS: Carbonate sand, ILL: Illite, BF2: Basalt F2, VCG: Volcanic glass, KAO: Kaolinite, BSS: Basalt sand, SCS: Siliciclastic sand. All error bars indicate the SD.

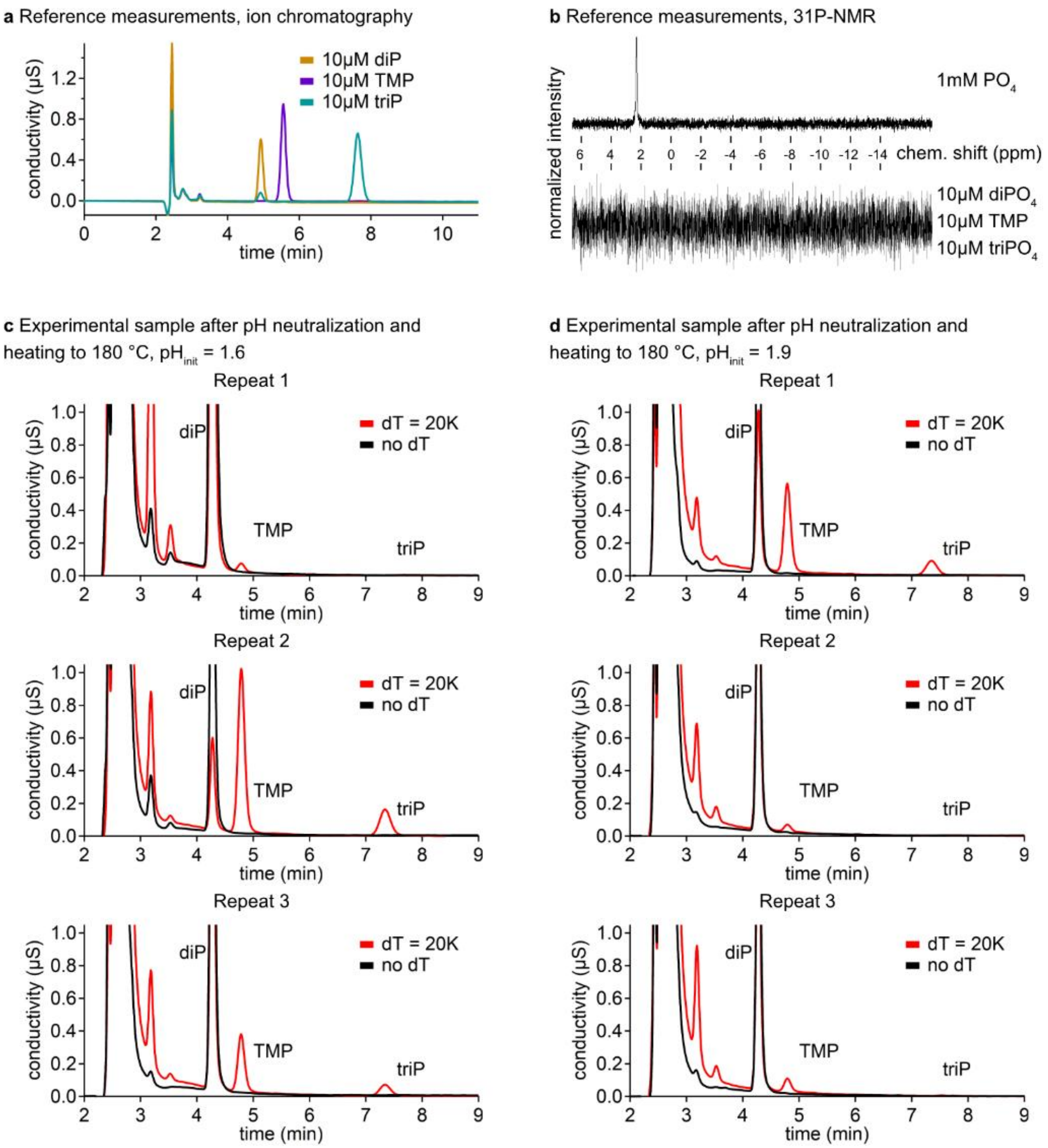

Supplementary Figure 11. Detection of polyphosphates

(a) Detection of 10 µM of diphosphate, trimetaphosphate and triphosphate. $Na_3P_3O_9$ and $Na_5P_3O_{10}$ were purchased from Sigma Aldrich (USA), $Na_4P_2O_7$ from CarlRoth GmbH (Germany). **(b)** Measurement of (upper) mixture of 10 µM diphosphate, trimetaphosphate and triphosphate and (lower) 1mM orthophosphate measured with 31P-NMR. **(c-d)** IC-chromatograms of samples after pH neutralization and heating to 180 °C. Samples from heat-flow-fractionated experiments are shown in red, bulk samples in black. Products are referenced in chromatograms. Large deviations between repeats are expected due to the multi-stage process of dissolution, heat-flow-driven fractionation, pH neutralization, heating and redissolution, of which, e.g., the heat-flow-driven fractionation is exponentially dependent on the applied temperature gradient[3].

Supplementary Tables

Name	# of point measurements	CaO / P_2O_5 (average)	CaO / P_2O_5 (SD)
Apatite Brazil	17	1.40	0.02
Apatite Canada	6	1.33	0.02
Apatite Mexico	10	1.31	0.02
Experimental (in, initial pH 1.9, replicate 1)	21	1.53	0.19
Experimental (bottom, initial pH 1.9, replicate 1)	7	1.38	0.16
Experimental (in, initial pH 1.9, replicate 3)	5	1.52	0.05
Experimental (bottom, initial pH 1.9, replicate 3)	13	1.39	0.29
Experimental (in, initial pH 3.6)	65	1.21	0.07
Experimental (bottom, initial pH 3.6)	64	1.39	0.15

Supplementary Table 1. CaO/P_2O_5 ratios of precipitates in re-neutralized samples.

Measurements were performed as described in the Methods. We assume errors increased due to small amounts of sample material and the presence of hydroxides, as shown before [4,5].

#M	Mate-rial	Na (%)	Mg (%)	Al (%)	Si (%)	P (%)	S (%)	K (%)	Ca (%)	Ti (%)	Mn (%)	Fe (%)
24	SCS	2.45	0.06	10.5	79.2	0.23	0.02	2.52	1.61	0.10	0.03	2.98
25	ILL	0.16	3.56	23.3	50.1	0.37	0.04	5.52	8.71	0.72	0.10	6.84
29	CAS	0.65	1.79	0.37	0.00	0.05	0.70	0.05	96.2	0.03	0.02	0.05
32	KAO	0.03	0.15	42.7	54.4	0.44	0.07	0.82	0.09	0.29	0.02	0.51
30	BSS	4.07	2.43	19.7	60.8	0.07	0.04	1.96	5.23	0.33	0.10	4.80
27	VCG	3.61	3.51	14.4	49.8	0.49	0.08	0.97	8.92	4.17	0.19	13.6
23	MON	0.21	1.35	15.7	77.3	0.03	0.03	2.53	0.18	0.33	0.02	1.87
32	ZEO	0.24	0.89	12.9	77.2	0.02	0.03	3.49	2.83	0.22	0.01	1.83

Supplementary Table 2. Composition of geomaterials tested for phosphate leaching and phosphate polymerization.

Measurements were performed as described in the Methods using EDX, the corresponding SEM images are shown in Supplementary Figs. 7-8. #M describes the number of point measurements. SCS: Siliciclastic sand, ILL: Illite, CAS: Carbonate sand, KAO: Kaolinite, BSS: Basalt sand, VCG: Volcanic glass, MON: Montmorillonite, ZEO: Zeolite.

Supplementary references

1. Baaske, P. et al. Extreme accumulation of nucleotides in simulated hydrothermal pore systems. Proc. Natl. Acad. Sci. **104**, 9346–9351 (2007).
2. Keil, L. M. R., Möller, F. M., Kieß, M., Kudella, P. W. & Mast, C. B. Proton gradients and pH oscillations emerge from heat flow at the microscale. Nat. Commun. **8**, 1897 (2017).
3. Matreux, T., Aikkila, P., Scheu, B., Braun, D. & Mast, C. B. Heat flows enrich prebiotic building blocks and enhance their reactivity. Nature **628**, 110–116 (2024).
4. Kleine-Boymann, M. et al. Discrimination between biologically relevant calcium phosphate phases by surface-analytical techniques. Appl. Surf. Sci. **309**, 27–32 (2014).
5. Miculescu, F. et al. Considerations and Influencing Parameters in EDS Microanalysis of Biogenic Hydroxyapatite. J. Funct. Biomater. **11**, 82 (2020).
