# Supplementary material for: Heat flows solubilize apatite to boost phosphate availability for prebiotic chemistry
Source: Nat Commun. 2025 Feb 20;16:1809. doi: 10.1038/s41467-025-57110-3 (PMC11842809; doi:10.1038/s41467-025-57110-3)
Supplement: Supplementary file 2 — Description of Additional Supplementary Files [file 41467_2025_57110_MOESM2_ESM.pdf]

Description of Additional Supplementary Files:

Supplementary Data 1: Python script using a PHREEQC implementation to calculate precipitation equilibria of acidic apatite solutions after repeated addition of sodium hydroxide as used in Fig. 2 and Table 2, with more details shown in Supplementary Fig. 4.
